# Inspiratory muscle strength training for lowering blood pressure and improving endothelial function in postmenopausal women: comparison with “standard of care” aerobic exercise

**DOI:** 10.3389/fphys.2022.967478

**Published:** 2022-08-29

**Authors:** Daniel H. Craighead, Kaitlin A. Freeberg, Narissa P. McCarty, Matthew J. Rossman, Kerrie L. Moreau, Zhiying You, Michel Chonchol, Douglas R. Seals

**Affiliations:** ^1^ Integrative Physiology of Aging Laboratory, Department of Integrative Physiology, University of Colorado Boulder, Boulder, CO, United States; ^2^ Division of Geriatric Medicine, University of Colorado Anschutz Medical Campus, Aurora, CO, United States; ^3^ Veterans Affairs Eastern Colorado Geriatric Research, Educational and Clinical Center, Denver, CO, United States; ^4^ Division of Renal Diseases and Hypertension, University of Colorado Anschutz Medical Campus, Aurora, CO, United States

**Keywords:** aging, IMST, time-efficient, cardiovascular disease, nitric oxide, oxidative stress

## Abstract

**Background:** High blood pressure (BP), particularly systolic BP (SBP), is the major modifiable risk factor for cardiovascular diseases and related disorders of aging. SBP increases markedly with aging in women such that the prevalence of above-normal SBP (i.e., ≥120 mmHg) in postmenopausal women exceeds rates in age-matched men. This increase in SBP is associated with vascular endothelial dysfunction, mediated by excessive reactive oxygen species-induced oxidative stress and consequent reductions in nitric oxide bioavailability. Moderate-intensity aerobic exercise is a recommended lifestyle strategy for reducing SBP. However, adherence to aerobic exercise guidelines among postmenopausal women is low (<30%) and aerobic exercise does not consistently enhance endothelial function in estrogen-deficient postmenopausal women. High-resistance inspiratory muscle strength training (IMST) is a time-efficient, adherable lifestyle intervention that involves inhaling against resistance through a handheld device (30 breaths/day). Here, we present the protocol for a randomized controlled trial investigating the efficacy of 3 months of high-resistance IMST compared to guideline-based, “standard-of-care” aerobic exercise training for decreasing SBP and improving endothelial function in estrogen-deficient postmenopausal women with above-normal SBP (120–159 mmHg) at baseline (ClinicalTrials.gov Identifier: NCT05000515).

**Methods:** A randomized, single-blind, parallel-group design clinical trial will be conducted in 72 (36/group) estrogen-deficient postmenopausal women with above-normal SBP. Participants will complete baseline testing and then be randomized to either 3 months of high-resistance IMST (30 breaths/day, 6 days/week, 75% maximal inspiratory pressure) or moderate-intensity aerobic exercise training (brisk walking 25 min/day, 6 days/week, 40–60% heart rate reserve). Outcome measures will be assessed after 3 months of either intervention. Following end-intervention testing, participants will abstain from their assigned intervention for 6 weeks, after which BP and endothelial function will be assessed to evaluate the potential persistent effects of the intervention on the primary and secondary outcomes.

**Discussion:** This study is designed to compare the effectiveness of time-efficient, high-resistance IMST to guideline-based aerobic exercise training for lowering SBP and improving endothelial function, and interrogating potential mechanisms of action, in estrogen-deficient postmenopausal women.

**Clinical Trial Registration:** ClinicalTrials.gov, Identifier: NCT05000515.

## Introduction

### Background and rationale

Above-normal casual (resting) blood pressure (BP), defined as systolic BP (SBP) ≥120 mmHg and/or diastolic BP (DBP) ≥80 mmHg (Whelton et al., 2018), increases the risk of developing cardiovascular diseases (CVD) and other chronic health conditions (Kivipelto et al., 2001; Guo et al., 2013; Huang et al., 2013, 2014).

In adults aged 50 years and older, the disease risks of high BP are driven primarily by above-normal SBP, as DBP plateaus at midlife and then actually decreases at older ages (Franklin et al., 1997). Absolute SBP levels are lower in premenopausal women compared to age-matched men. However, SBP increases more rapidly in women after the menopausal transition, such that the prevalence of above-normal SBP in postmenopausal women meets and then exceeds rates observed in age-matched men (Ji et al., 2020; Tsao et al., 2022).

This greater increase in SBP in postmenopausal women plays a key role in the increased risk of CVD, including stroke, heart failure with preserved ejection fraction, and other cardiovascular complications in older women compared to men (Beale et al., 2018; Tsao et al., 2022).

Currently, >75% of postmenopausal women in the United States have above-normal SBP (Abramson et al., 2018; Whelton et al., 2018) and the number of postmenopausal women is expected to grow by 37% by the year 2060 (United States Census Bureau, 2017). In addition, established treatments for lowering and controlling SBP are less effective in postmenopausal women compared to men (Lloyd-Jones et al., 2005; Pimenta, 2012). Thus, establishing novel strategies for lowering SBP in postmenopausal women is a biomedical research priority.

Increasing SBP is closely linked to impaired vascular endothelial function (Buford, 2016). Compared to their normotensive peers, postmenopausal women with above-normal SBP demonstrate impaired endothelium-dependent dilation (Craighead et al., 2020), an independent predictor of CVD (Inaba et al., 2010; Green et al., 2011; Ras et al., 2013; Xu et al., 2014; Matsuzawa et al., 2015). This endothelial dysfunction is driven by oxidative stress-mediated suppression of the bioavailability of nitric oxide (NO), a critical vasodilatory molecule (Doshi et al., 2001; Seals et al., 2011).

SBP-lowering therapies that concomitantly improve vascular endothelial function are superior for reducing CVD risk compared to therapies that have isolated effects on SBP (Modena et al., 2002; Kitta et al., 2009). Therefore, interventions that both lower SBP and improve vascular endothelial function hold strong promise for disease prevention in postmenopausal women.

Among other health benefits, regular moderate-intensity aerobic exercise is a recommended lifestyle intervention for lowering SBP (Whelton et al., 2018). However, only 25–30% of postmenopausal women in the United States meet guidelines for 150 min/week of moderate-intensity aerobic exercise (Keadle et al., 2016; Bennie et al., 2019). The most commonly cited barrier contributing to this low rate of adherence is lack of time (Stutts, 2002; El Ansari and Lovell, 2009; Kelly et al., 2016). Other common barriers include facility access, mobility issues, and financial cost (Yarwood et al., 2005; Siddiqi et al., 2011; Babakus and Thompson, 2012; Kelly et al., 2016).

Along with SBP-lowering effects, aerobic exercise also improves vascular endothelial function in midlife/older men (DeSouza et al., 2000; Pierce et al., 2011b), but does not consistently induce the same benefits in estrogen-deficient postmenopausal women (i.e., women not taking hormone therapy) (Pierce et al., 2011b; Moreau et al., 2013; Santos-Parker et al., 2017), a group that makes up over 90% of the postmenopausal women in the United States (Connelly et al., 2000).

This is due to the inability of aerobic exercise to reduce oxidative stress in women deplete of estrogen (Moreau et al., 2013). Estrogen repletion through hormone therapy prior to training restores the beneficial effects of aerobic exercise on the vascular endothelium in postmenopausal women (Moreau et al., 2013). However, hormone therapy is contraindicated in most cases and not a viable option for enhancing the effects of aerobic exercise in postmenopausal women (Rossouw et al., 2002). As such, developing novel lifestyle interventions that promote adherence, lower SBP, and improve endothelial function in estrogen-deficient postmenopausal women is a biomedical research priority (Seals et al., 2019).

One such potential intervention is high-resistance inspiratory muscle strength training (IMST), which involves breathing in against resistance through a handheld device (Craighead et al., 2019). In contrast to conventional inspiratory muscle training against low-to-moderate levels of resistance performed for extended durations, high-resistance IMST utilizes a low number of repetitions against a near-maximal resistance, resulting in a daily time commitment of only approximately 5 min, overcoming the critical time-availability barrier (Craighead et al., 2021a). Additionally, by using an affordable, portable device, high-resistance IMST overcomes many common barriers associated with aerobic exercise and holds promise for future public health translation (Craighead et al., 2022). However, the efficacy of high-resistance IMST for inducing cardiovascular health benefits, particularly in estrogen-deficient postmenopausal women, needs to be firmly established.

We recently completed a pilot study in midlife/older men and women (aged 50–79 years) with above-normal initial SBP comparing 6 weeks of high-resistance IMST (30 breaths/day, 6 days/week, 75% maximal inspiratory pressure [PI_MAX_]) to low-resistance (15% PI_MAX_) sham control training. IMST was safe (no serious adverse events), tolerable (no dropouts due to adverse events) and promoted adherence (94% of prescribed training sessions performed) (Craighead et al., 2021b).

In this pilot trial, IMST also induced clinically meaningful improvements in cardiovascular function. Casual SBP was reduced by 9 mmHg and this reduction was ∼75% sustained 6 weeks after completing the last IMST training session; casual DBP also was reduced by 2 mmHg. Twenty-four-hour SBP, a CVD risk factor independent of casual SBP (Clement et al., 2003; Dolan et al., 2005), decreased by 4 mmHg. Brachial artery flow-mediated dilation (FMD_BA_), a measure of endothelial function, was increased by approximately 45%. Improvements in cardiovascular function were associated with reductions in oxidative stress and increased NO bioavailability (Craighead et al., 2021b).

Although establishing the sex-specific effects of IMST was not a primary objective of our trial due to a limited sample size, our initial results suggested IMST was at least equally as effective for lowering SBP and improving endothelial function in estrogen-deficient postmenopausal women as in age-matched men (Craighead et al., 2021b).

**FIGURE 1:**
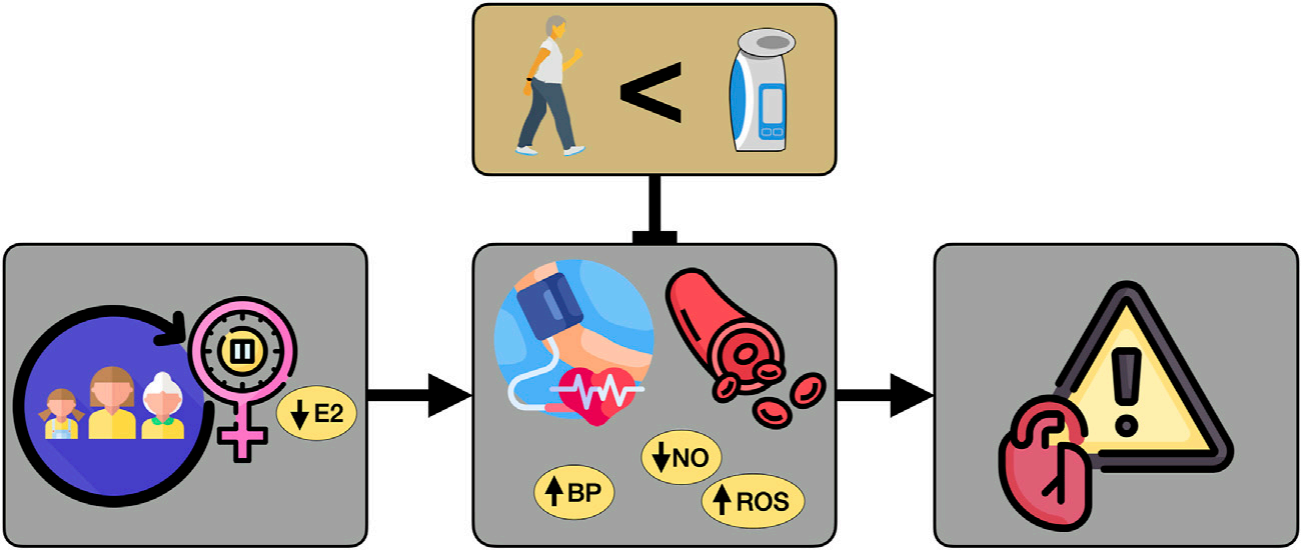
Hypothesis. Hypothesized effects of high-resistance inspiratory muscle strength training for lowering systolic blood pressure and improving endothelial function to a greater extent than guideline-based moderate-intensity aerobic exercise in estrogen-deficient postmenopausal women. E2, estrogen; BP, blood pressure; NO, nitric oxide; ROS, reactive oxygen species.

To extend these encouraging preliminary results from our pilot trial and establish the efficacy of high-resistance IMST in estrogen-deficient postmenopausal women, we are conducting a randomized, single-blind, parallel group design clinical trial seeking to investigate the comparative effectiveness of high-resistance IMST versus moderate-intensity aerobic exercise for 1) lowering casual and 24-h SBP; 2) improving vascular endothelial function; and 3) beneficially modulating oxidative stress and NO bioavailability in estrogen-deficient postmenopausal women with initial above-normal SBP (120–159 mmHg) (Figure 1).

We also will further assess the safety and tolerability of, and adherence to, the interventions. Potential persistent effects of the interventions on cardiovascular function following abstention also will be examined.

We will address these aims by assessing our outcome measures before and at the end of 3 months of high-resistance IMST (30 breaths/day, 6 days/week, 75% PI_MAX_) or guideline-based aerobic exercise (150 min/week brisk walking), and 6 weeks after stopping the interventions, in estrogen-deficient postmenopausal women with above-normal initial SBP.

## Methods

### Study design

This is a randomized, single-blind, parallel-group design clinical trial assessing 3 months of high-resistance IMST versus moderate-intensity aerobic exercise (brisk walking) in 72 (36/group) estrogen-deficient postmenopausal women with above-normal SBP at baseline. The intervention duration for the current protocol was chosen based on clinical guidelines for lifestyle-based treatment of high BP, which recommend 3 months of healthy lifestyle practices as a first-line intervention for lowering BP (Whelton et al., 2018).

Postmenopausal women with SBP 120–159 mmHg who have not taken hormone therapies within the previous 12 months will be eligible to participate provided they meet all criteria for inclusion and exclusion (Table 1).

**TABLE 1:** **|** Inclusion and exclusion criteria.

Inclusion criteria	Exclusion criteria
• Postmenopausal women (>12 months of amenorrhea)	• Early menopause (menopause before age 45 years)
• Estrogen deficient (no hormone therapies within the previous 12 months)	• Having had a hysterectomy or oophorectomy
• Age 50 years or older	• Current smoker
• Ability to provide informed consent	• Chronic overt medical condition (e.g., recent myocardial infarction or stroke, cancer, diabetes)
• Willing to accept random assignment to condition	• Alcohol abuse or dependence
• SBP 120–159 mmHg	• Uncontrolled thyroid disease or change in thyroid medication within previous 3 months
• Body mass index <40 kg/m^2^	• History of uncontrolled hypertension (SBP >180 mmHg and/or DBP >120 mmHg)
• Weight stable in the prior 3 months (<2 kg weight change) and willing to remain weight stable throughout the study	• Abnormal BP response to exercise (drop in SBP below resting pressure or SBP >260 mmHg or DBP >115 mmHg)
• Absence of clinical disease as determined by medical history, physical examination, blood chemistries, ankle-brachial index, and 12-lead ECG	• Regular vigorous aerobic/endurance exercise (>4 bouts/week, >30 min/bout at a workload >6 METS)
Ankle-brachial index >0.7	
Total cholesterol <240 mg/dl	• Blood donation within 8 weeks prior to enrolling in the study; unwilling to abstain from donating blood for 8 weeks after completing the study
Fasting plasma glucose <126 mg/dl	
Normal 12-lead ECG at rest and during graded treadmill exercise to fatigue	
• Subjects taking antihypertensive medications will be included provided they meet the other inclusion criteria, including SBP. Medication regimen (prescription and dosing) must be stable for at least 3 months prior to enrollment in the study and must remain stable during the study	

SBP, systolic blood pressure; DBP, diastolic blood pressure; ECG, electrocardiogram; METs, metabolic equivalents.

### Outcomes

All outcomes will be measured before and after 3 months of high-resistance IMST or moderate-intensity aerobic exercise. Casual SBP, 24-h SBP and FMD_BA_ also will be measured 6 weeks after cessation of IMST and aerobic exercise to determine the long-lasting effects of IMST on our primary and secondary outcome measures.

All cardiovascular measurements and blood sampling will be performed after a >5-h fast from food (water allowed) and caffeine, >24-h abstention from alcohol and vigorous physical activity, and >48-h abstention from dietary supplements and over-the-counter medications. Supervised training sessions and check-in visits will be performed without dietary, medication, or activity restrictions.

### Primary outcome

#### Casual (resting) systolic blood pressure

Casual (resting) SBP will be measured and classified according to 2017 American College of Cardiology/American Heart Association guidelines (Whelton et al., 2018). Subjects will rest quietly for at least 5 min while seated with their back supported, feet flat on the floor, and arm at heart level. BP will be measured in triplicate, with a 2-min recovery between each measure, over the brachial artery of the non-dominant arm via automated oscillometric sphygmomanometer (Mindray), validated according to the Association for the Advancement of Medical Instrumentation standards (Anwar et al., 1997) and regularly calibrated by the manufacturer.

**FIGURE 2:**
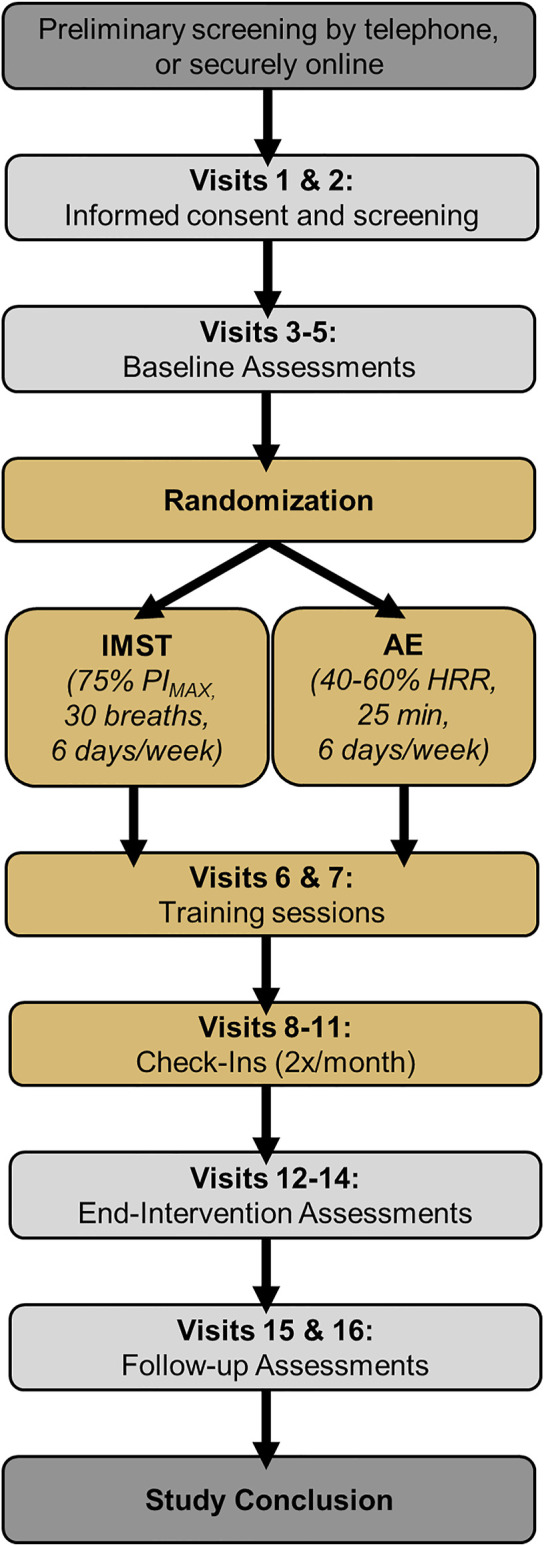
Study design. IMST, inspiratory muscle strength training; AE, aerobic exercise; PI_MAX_, maximal inspiratory pressure.

During baseline, end-intervention, and follow-up testing, casual BP will be measured on two separate days, >24 h apart, and casual SBP and DBP will be defined as the average over the 2 days. Casual SBP and DBP also will be measured at a single timepoint during each supervised training session and check-in visit (Figure 2) to monitor changes in BP across the intervention.

### Secondary outcomes

#### 24-Hour ambulatory systolic blood pressure

Brachial artery BP will be measured every 20 min during the day (16-h period) and every 60 min at night (8-h period) over 24 h according to each participant’s individual self-reported sleep-wake cycle.

BP will be measured via ambulatory BP monitoring (Oscar 2, SunTech Medical), validated according to British Hypertension Society standards (Goodwin et al., 2007). Participants will be outfitted with the ambulatory BP monitor by a trained investigator and given written and verbal instructions regarding monitor operation. The BP cuff will be placed securely over the non-dominant arm. Participants also will be given a diary to record physical activity or psychological events that occur over the 24-h period that may influence BP measurements. Ambulatory BP recordings will be analyzed for mean 24-h, daytime, and nighttime SBP and DBP.

#### Vascular endothelial function

FMD_BA_, i.e., endothelium-dependent dilation to a flow stimulus, is the “gold standard” non-invasive measure of conduit artery NO-mediated endothelial function (Doshi et al., 2001; Seals et al., 2011). FMD_BA_ will be determined using high-resolution ultrasonography (Canon Xario 200) and analyzed with a commercially available software package (Vascular Analysis Tools 5.10.9, Medical Imaging Applications, LLC). Brachial artery diameter and blood flow velocity will be measured at baseline and for 2 min following reactive hyperemia induced by 5 min of forearm blood flow occlusion (upper forearm cuff placement). Data will be expressed both as a percent and absolute (millimeter) change in brachial artery diameter from baseline (pre-cuff inflation diameter). Peak and area under the curve shear rate will be calculated, and if group- or condition-differences exist, FMD_BA_ will be adjusted accordingly (Harris et al., 2010). Endothelium-independent dilation will be determined by measuring brachial artery dilation for 10 min after sublingual nitroglycerin (0.4 mg dissolvable tablet) to assess vascular smooth muscle sensitivity to NO (Seals et al., 2011).

### Other outcomes

#### Oxidative stress-associated suppression of endothelial function

Tonic suppression of endothelial function by oxidative stress will be assessed by measuring the improvement in FMD_BA_ from baseline (saline control) in response to acute (intravenous) infusion of supraphysiological concentrations of vitamin C, a potent antioxidant that scavenges reactive oxygen species (ROS) (Eskurza et al., 2004; Walker et al., 2012; Jablonski et al., 2013). A bolus dose of 0.06 g vitamin C/kg fat-free mass dissolved in 100 ml of saline will be infused intravenously at a rate of 5 ml/min for 20 min, followed immediately by a “drip-infusion” of 0.02 g/kg fat-free mass dissolved in 30 ml of saline infused at a rate of 0.5 ml/min. FMD_BA_ will be performed as described above at the end of the 20-min bolus during the “drip infusion,” when peak levels of vitamin C occur (Eskurza et al., 2004).

#### Endothelial cell protein abundance

Endothelial cell markers of oxidative modification of proteins (nitrotyrosine abundance), pro-oxidant signaling (NADPH oxidase subunit p47^phox^) and antioxidant defenses (manganese superoxide dismutase) will be assessed using methods established in our laboratory (Donato et al., 2008; Pierce et al., 2011a; Jablonski et al., 2011). Endothelial cells will be collected from an antecubital vein by a trained nurse using 2 sterile 0.025-inch J-wires briefly advanced through an intravenous catheter. Cells will be recovered by centrifugation, fixed with formaldehyde, and slides will be prepared and frozen for later analysis.

Slides will be stained for the primary antibody of interest and a complementary fluorescent secondary Alexafluor 647 antibody (Invitrogen). Slides will also be stained for positive identification of endothelial phenotype and with DAPI for nuclear integrity. Images will be captured digitally and analyzed using Image J software (NIH). Values will be reported as ratios of subject endothelial cell protein expression to human umbilical vein endothelial cell (HUVEC) control protein expression to remove the possible confound of differences in intensity of staining between sessions. Importantly, we have shown that endothelial cells sampled from peripheral veins provides the same information as arterial sampling without the need for invasive brachial artery catheterization (Silver et al., 2010).

#### Endothelial cell nitric oxide and reactive oxygen species production with serum exposure

In order to determine the role of circulating factors in modulating endothelial cell function, NO and ROS production will be measured in HUVECs treated with serum obtained from subjects before versus after 3 months of high-resistance IMST or aerobic exercise as previously described (Craighead et al., 2021b). HUVECs (PromoCell; used after 2–4 passages) will be plated in 96-well culture plates and incubated with basal media supplemented with 10% subject serum under standard conditions (37°C, 5% CO_2_) for 24 h. Following incubation, cells will be co-incubated with fluorescent dye mixture (DAR-4M AM and CellROX Deep Red). Fluorescent imaging of cytoplasmic DAR-4M AM before and 5 min after addition of 100 µM acetylcholine will quantify the capacity of HUVECs to generate NO while basal CellROX fluorescent intensity will quantify intracellular ROS levels. Analysis will be done with Celleste (Invitrogen) and data expressed as a fold change from NO or ROS production with baseline (pre-intervention) serum exposure.

To assess the specific circulating factors involved in mediating changes in endothelial cell function, HUVECs also will be cultured with plasma metabolites that change significantly with IMST or aerobic exercise training (identification of these metabolites described below in Targeted Plasma Metabolomics). The HUVECs will be cultured as described above with and without identified metabolites administered at estimated physiological concentrations observed after IMST or aerobic exercise, and NO and ROS production will be measured.

#### Targeted plasma metabolomics

We will use targeted plasma metabolomics to identify circulating factors contributing to potential changes in endothelial cell NO and ROS production with IMST or aerobic exercise. Subject plasma obtained before and after the IMST and aerobic exercise interventions will be analyzed as described previously (Gehrke et al., 2017). Briefly, plasma samples will be thawed on ice. A 20 µL aliquot of plasma will be diluted with 480 µl of ice-cold methanol/acetonitrile/water (5/3/2). Extractions will be performed as previously described (Nemkov et al., 2015). Supernatants from the extraction will be analyzed with a 5-min C18 gradient on an ultra-high-performance liquid chromatograph (Thermo Fisher Vanquish) coupled with a mass spectrometer (Thermo Fisher Q Exactive). Raw files will be converted to mzXML format (RawConverter, Yates Laboratory, The Scripps Research Institute). Metabolite assignments will be made using Maven (Princeton, NJ).

#### Safety and tolerability

Safety, i.e., risk to the subject, of both treatment arms will be assessed by recording adverse events during the intervention. Tolerability, i.e., the degree to which overt adverse events are tolerated by participants, will be determined based on the number of participants that drop out of the study due to adverse events.

#### Adherence

Adherence will be determined as the proportion of prescribed IMST or aerobic exercise training sessions that were performed appropriately, i.e., correct duration and intensity. In the IMST group, adherence will be determined from the training session information saved on the internal data storage of the POWERBreathe K3 training device. In the aerobic exercise group, adherence will be assessed through heart rate data stored on the Polar OH1 heart rate monitor.

### Measurements related to lifestyle behaviors and body weight control

The following measures will be made at baseline and end-intervention to account for subject characteristics known to impact cardiovascular function. Physical activity will be monitored objectively via accelerometry (GENEActiv; 3-day recording) (Matthews et al., 2002) and subjectively via the Community Healthy Activities Model Program for Seniors questionnaire (Stewart et al., 2001) to ensure participant physical activity remains unchanged after versus before the intervention. Maximal oxygen consumption using indirect calorimetry during incremental treadmill exercise (Balke protocol) will be measured to document aerobic fitness (DeSouza et al., 2000). Body composition will be assessed by dual energy x-ray absorptiometry (DEXA) and anthropometry. Body mass also will be assessed during supervised training sessions and check-in visits (Visits 6-11, Figure 2).

### Selection of subjects and intervention administration

#### Study location and timeline

All study visits will be performed at the Clinical Translational Research Center (CTRC) at the University of Colorado Boulder Campus. We plan to recruit 90 subjects to account for an approximate 20% dropout rate to achieve our goal of completing testing on 72 participants.

#### Recruitment and eligibility

Recruitment efforts will include newspaper and social media advertisements; University of Colorado Boulder campus email bulletins sent to faculty, staff, and students; and flyers posted in public locations or handed out at senior centers and community fairs in the greater Boulder/Denver area. Interested individuals who contact the laboratory via telephone or email will be directed to fill out a general screening form (either over the phone or online) to determine their eligibility for the study. Interested individuals also can fill out the general screening form through a link provided on the laboratory website. Potential participants who meet the initial inclusion/exclusion criteria based on the general screening form will be contacted and scheduled for informed consent and screening visits. Individuals who do not meet inclusion/exclusion criteria also will be notified. Individuals will be allowed to ask any questions they have about the study at any point in the pre-consent process. All email, phone, and/or mail communication with potential participants will be kept confidential.

#### Informed consent and screening

Informed consent will be obtained in-person by a trained and approved member of the study team. The participant will be given an overview of the study and all study procedures, and have time to read through the informed consent document and invited to ask any questions. Both the participant and the investigator obtaining consent will sign the consent form and a copy of the signed form will be given to the participant. All study procedures have been reviewed and approved by the Institutional Review Board at the University of Colorado Boulder (Protocol #21-0489).

Following informed consent, participants will undergo screening assessments to further determine eligibility based on meeting enrollment criteria (Visits 1-2, Figure 2). Participants will be asked to abstain from food and caffeine for 5 h, alcohol and strenuous exercise for 24 h, and dietary supplements and non-prescription medications for 48 h before the screening visits. Screening assessments will include:

• Resting BP and heart rate
• Height and body weight
• Medical history and physical examination
• Exercise stress test
• Ankle-brachial index
• Questionnaire and paperwork
• Family medical history
• Physical activity *via* the Modifiable Activity Questionnaire (Kriska et al., 1990; Vuillemin et al., 2000)
• Screening blood collection

Each participant’s results from screening will be reviewed by the study medical director, who will approve each individual’s safety for participating in the clinical trial.

#### Baseline assessments

Eligible participants who qualify for the study based on screening visits will undergo baseline experimental testing within a ≤10-day period (Visits 3-5, Figure 2). Participants will be asked to avoid major lifestyle changes for the duration of the study, including changes in physical activity, diet, and body weight, unless the changes are deemed necessary by the subject’s personal physician. Subjects will be asked to maintain a stable medication regimen through the study and to notify the study team should their physician recommend an immediate change to their medication prescriptions (change in medications or dosing).

#### Randomization and Blinding

After baseline assessments, participants will be randomized to 3 months of high-resistance IMST or aerobic exercise training. Block randomization will be employed based on age (≤64 years versus ≥65 years) and baseline SBP (elevated BP/stage 1 hypertension: 120–139 mmHg versus stage II hypertension: ≥140 mmHg) (Whelton et al., 2018). Randomization will be performed by a professional research assistant not involved with data analysis who will remain unblinded for the duration of the study. Investigators responsible for collection and analysis of outcome measures will be blinded to participant group assignment. Given the design of this study comparing different lifestyle interventions, participant blinding is not possible.

#### Intervention

##### High-resistance inspiratory muscle strength training

Participants in the high-resistance IMST group will train using the POWERBreathe K3 tapered-resistance inspiratory training device. Individual training sessions will entail performing 30 inspiratory maneuvers, consisting of 5 sets of 6 inspirations with a 1-min rest between sets. Training sessions will be performed 6 days per week for 3 months. Subjects will continue to train during the end-intervention testing period (described below) to maintain the training effect.

Inspiratory resistance on the POWERBreathe K3 will be set to 55% of each individual’s PI_MAX_ during week 1 of training, 65% of PI_MAX_ during weeks 2 and 3 of training, and then at 75% of PI_MAX_ throughout the remainder of the intervention. PI_MAX_ will be reassessed during supervised training sessions and check-in visits and the absolute training intensity adjusted to account for improvements in inspiratory muscle function throughout the intervention.

The first IMST training session will be supervised in the laboratory by an unblinded research assistant to train the participant on proper IMST technique (Visit 6, Figure 2). A second IMST training session will be supervised 1 week later to ensure maintenance of the proper technique (Visit 7, Figure 2). All other training sessions will be performed unsupervised, but with training documented by the internal storage of the POWERBreathe K3 training device.

PI_MAX_ will be assessed at baseline, during check-in visits (Visits 8-11, Figure 2) and during end-intervention testing to maintain an appropriate training stimulus and document changes in inspiratory muscle strength. To calculate PI_MAX_ subjects will perform a series of maximal inspiratory efforts against a near-infinite resistance using the POWERBreathe KH2 device and associated Breathlink software. The average of the 3 largest pressures generated will be calculated as PI_MAX_ (Kellerman et al., 2000; Vranish and Bailey, 2015). PI_MAX_ also will be measured at baseline and end-intervention in the moderate-intensity aerobic exercise group to allow for comparisons between groups.

##### Moderate-intensity aerobic exercise training

Subjects will walk at an intensity that elicits a heart rate between 40% and 60% of heart rate reserve, calculated as described previously (Tanaka et al., 1998). Resting heart rate will be measured during seated casual BP measurements and maximal heart rate will be determined from the maximal aerobic exercise test performed at baseline. Subjects will walk for 25 min per day, 6 days per week to meet aerobic exercise guidelines for 150 min/week of moderate-intensity exercise (Piercy et al., 2018) and to match the training frequency of the IMST group. During the aerobic exercise training sessions, a validated Polar OH1 heart rate monitor (Hettiarachchi et al., 2019) will provide real-time feedback to participants.

The first training session will take place on the laboratory treadmill, supervised by a research assistant, to familiarize the subjects with the appropriate exercise intensity and heart rate monitor use (Visit 6, Figure 2). A second aerobic exercise training session will be supervised 1 week later to confirm proper execution of training and to match subject contact time with the IMST group (Visit 7, Figure 2). All other sessions will be performed on-their-own, either outdoors or on a treadmill, with adherence documented by the heart rate monitor and recorded by a study investigator during check-in visits (Visits 8-11, Figure 2).

#### End-intervention assessments

After completing 3 months of either high-resistance IMST or aerobic exercise training, participants will return to the University of Colorado Boulder CTRC for reassessment of all outcome measures within a ≤10-day period (Visits 12-14, Figure 2). Participants will continue their assigned intervention throughout end-intervention testing to maintain the training stimulus. However, experimental testing will be scheduled to occur 24–48 h following the most recent training session to avoid any acute effects of the interventions. All end-intervention measurements will be identical to, and made under the same experimental conditions as, baseline testing.

#### Intervention abstention and follow-up testing

Once all end-intervention data have been collected, participants will return their training device (POWERBreathe K3 or Polar OH1 heart rate monitor) to the study staff and be instructed to halt their assigned intervention for a 6-week free-living period. During this 6-week period, participants will be asked not to make any lifestyle changes (outside of abstention from the study intervention). Participants will return to the laboratory 6 weeks later to perform follow-up testing for casual BP, 24-h BP, and FMD_BA_ (Visits 15 and 16, Figure 2) to determine the potential persistent effects of high-resistance IMST or moderate-intensity aerobic exercise on BP and vascular function (study primary and secondary outcomes).

#### Individual subject stopping criteria

Subjects will stop participation after successful completion of the study. Participants also can withdraw their informed consent at any time and for any reason. Investigators may also remove participants from the study at any time due to significant non-compliance with the protocol (i.e., procedures, assessments); any adverse event that, in the opinion of the medical director, indicates that continuing in the study is not in the best interest of the participant; loss of the participant’s ability to freely provide consent; or other serious changes in physical or mental health.

#### Adverse events

Participants will be instructed to inform members of the study team of any adverse events they experience while enrolled in the study. Mild and moderate adverse events will be regularly reported to the University of Colorado Boulder Institutional Review Board (IRB), National Institutes of Health, and the study data safety monitoring board. Serious adverse events will be reported to the study medical director within 24 h of the study team’s awareness of the adverse event and to the University of Colorado Boulder IRB within 5 days. An unanticipated adverse event which meets the University of Colorado Boulder IRB definition of an unanticipated problem (i.e., any unanticipated and undesirable effect arising from participation in research that results in an increased risk of harm or injury to a subject, or which suggests the possibility of increased risks to other subjects) will be reported to the University of Colorado Boulder IRB using the corresponding form within 5 days of occurrence.

#### Statistical design, power, and analysis plan

Sample size calculations and pre-determined statistical power for this randomized, single-blind, parallel group design clinical trial are based on the results from the estrogen-deficient postmenopausal women enrolled in our IMST pilot study (Craighead et al., 2021b) and results from a meta-analysis on the effects of aerobic exercise on SBP in our target population (Kelley, 1999).

Accordingly, it was estimated that aerobic exercise reduces casual SBP by 3.0 ± 3.7 mmHg (mean ± standard deviation), while high-resistance IMST reduces casual SBP by 7.6 ± 7.4 mmHg. With these parameters, it was estimated that 36 subjects per group (72 total) would provide 90% power to detect a difference in the change in casual SBP between groups with a two-sided type I error rate of *p* = 0.05. To account for a potential dropout rate of ∼20%, we will enroll 45 participants into each group (90 participants total).

Block randomization by age (midlife: ≤64 years; older ≥65 years) and baseline SBP (elevated SBP/stage 1 hypertension: 120–139 mmHg; stage 2 hypertension: ≥140 mmHg) will be employed. Data for all outcome variables will be collected at baseline and at the end of the respective 3-month interventions. In addition, casual BP, 24-h BP and FMD_BA_ will be collected after abstaining from the interventions for 6 weeks to assess potential persistent effects of previous training on these outcomes.

Descriptive statistics will be calculated for all baseline and outcome variables. Means and standard deviations will be calculated for all continuous variables, and frequency and proportion will be provided for all categorical variables. Data transformation will be performed if appropriate. The 95% confidence interval will be calculated as needed.

Repeated measures analysis by linear mixed effects models will be used to assess changes in casual and 24-h BP and FMD_BA_. Data from baseline, end-intervention and 6 weeks after completing the intervention will be included in the model. Based on this model, comparisons in the changes in BP between the two groups will be performed to assess the treatment effect. The basic model will include group, measurement timepoint, and the group by time interaction. Potential confounders such as time post menopause or use of antihypertensive medications can be included for adjustment purposes.

The same linear mixed effects model will be used to evaluate the intervention effect on our mechanistic outcomes at baseline and end-intervention. In addition, the association between the change in FMD_BA_ and changes in mechanistic probes will be assessed and linear regression model analysis used to adjust for potential confounders. Finally, mediation analysis will be used to provide evidence for a potential causal influence of our mechanistic probes for contributing to changes in FMD_BA_ with IMST or aerobic exercise (Mascha et al., 2013; Valente et al., 2017).

Adherence will be calculated as the percentage of prescribed training sessions performed appropriately and compared between groups with an unpaired t-test. Safety and tolerability will be described qualitatively.

#### Data management

All participant identities and records will be kept strictly confidential. Only the principal investigator and the study team will have access to identifiable data from this study. Physical data will be stored in locking filing cabinets within a secured-entry laboratory space. Electronic data will be stored on a laboratory server accessed by individual user sign-on and passwords, and through the Research Electronic Data Capture (REDCap) system, a secure, web-based data collection system. Identifiable subject information will not be presented in any publication arising from this study.

#### Data safety monitoring

This clinical trial is subject to oversight by an external data safety monitoring board, that is, completely independent of the study team. The board will meet twice per year to review safety and enrollment data. Additionally, a physician investigator will provide day-to-day medical oversight to ensure participant safety by serving as medical director on this protocol.

## Expected results and their interpretation

Based on the published results from our pilot study on high-resistance IMST (Craighead et al., 2021b) and previous aerobic exercise interventions in estrogen-deficient postmenopausal women (Pierce et al., 2011b; Moreau et al., 2013), we expect the following results in our study population of estrogen-deficient postmenopausal women with above-normal SBP at baseline.

**FIGURE 3:**
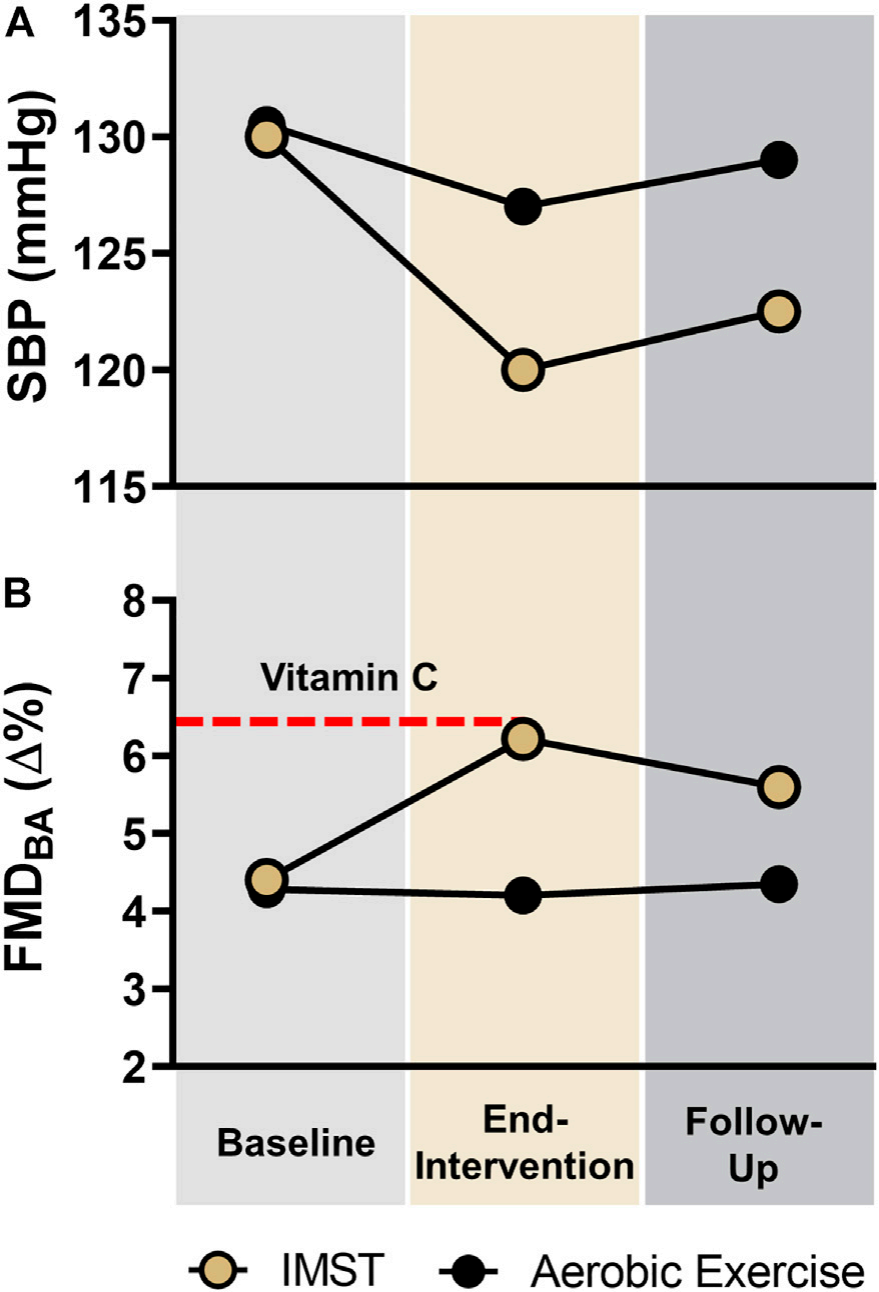
Expected Results. Expected results for **(A)** casual systolic blood pressure (SBP; primary outcome) and 24-h SBP (secondary outcome); and for **(B)** endothelial function measured by brachial artery flow-mediated dilation (FMD_BA_; secondary outcome) in the high-resistance inspiratory muscle strength training (IMST) and moderate-intensity aerobic exercise groups across the intervention. Dashed line represents expected FMD_BA_ with supratherapeutic infusion of the potent antioxidant, vitamin C, in both subject groups.

We expect a greater reduction in casual and 24-h SBP following 3 months of high-resistance IMST versus aerobic exercise training (Figure 3A). We also expect a greater percentage and absolute (mmHg) reduction in SBP to be present after 6 weeks of abstention from IMST versus aerobic exercise training (Figure 3A).

We also expect a greater improvement in FMD_BA_ following high-resistance IMST compared to aerobic exercise training, and that the improvement in FMD_BA_ following IMST will be at least partially sustained 6 weeks later (Figure 3B).

At end-intervention, we anticipate that high-resistance IMST will reduce or abolish the acute increase in FMD_BA_ observed after systemic infusion of vitamin C at baseline, but that aerobic exercise training will have no effect (Figure 3B). We expect that in biopsied endothelial cells, high-resistance IMST will decrease nitrotyrosine (marker of oxidative stress), decrease the expression of the oxidant enzyme NADPH oxidase, and increase expression of the antioxidant enzyme MnSOD to a greater extent than aerobic exercise training. We also expect endothelial cells cultured with subject serum obtained after (vs. before) IMST will increase NO and decrease ROS production more than serum obtained after (vs. before) aerobic exercise training. We also expect to identify changes in circulating metabolites with IMST that more greatly modulate endothelial cell NO and ROS production than aerobic exercise training does.

In addition, we expect to observe higher rates of adherence to the high-resistance IMST intervention compared to aerobic exercise training. Finally, we expect to observe fewer intervention-related adverse events and dropouts due to adverse events in the IMST group, suggesting greater safety and tolerability of IMST compared to aerobic exercise.

## Discussion

CVD is the leading cause of death worldwide (Tsao et al., 2022). CVD risk is increased in postmenopausal compared to premenopausal women, in part due to increases in SBP and vascular endothelial dysfunction which predominantly occur post menopause (Moreau et al., 2012, 2020; Whelton et al., 2018). Aerobic exercise is the most studied evidence-based intervention for improving cardiovascular health with aging. However, adherence to aerobic exercise guidelines is poor (Keadle et al., 2016; Bennie et al., 2019) and aerobic exercise is not consistently reported to improve endothelial function in estrogen-deficient postmenopausal women (Seals et al., 2019). Therefore, novel interventions that promote adherence and demonstrate efficacy for lowering SBP and improving endothelial function in this important population are needed.

This study protocol will assess the comparative effectiveness of 3 months of time-efficient, high-resistance IMST versus guideline-based, moderate-intensity aerobic exercise training for lowering SBP and improving vascular endothelial function in estrogen-deficient postmenopausal women with above-normal SBP at baseline. Our laboratory has successfully completed high-resistance IMST (Craighead et al., 2021b) and aerobic exercise intervention trials (DeSouza et al., 2000; Pierce et al., 2011b; Moreau et al., 2013), and performed all the experimental procedures to be employed in this study. Thus, we do not expect any significant problems implementing this protocol within our laboratory.

Through completion of this study, we will be able to determine whether 3 months of high-resistance IMST 1) lowers and sustains the reduction in casual and 24-h SBP; 2) improves and sustains the improvement in vascular endothelial function; and 3) is safe, tolerable, and associated with good adherence. We also will be able to gain insight into the potential mechanisms through which high-resistance IMST may improve cardiovascular function.

### Novelty and innovation

This study builds on promising findings from our pilot clinical trial that suggested high-resistance IMST may be able to lower SBP and improve endothelial function in a small subset (*n* = 7) of estrogen-deficient postmenopausal women. The larger sample size employed in this properly powered clinical trial will enable us to definitively determine the efficacy of high-resistance IMST in this important population.

The intervention duration for the current protocol was chosen based on clinical guidelines for lifestyle-based treatment of high BP, which recommend 3 months of healthy lifestyle practices as a first-line intervention for lowering BP (Whelton et al., 2018). This intervention duration also will extend the findings from our previous pilot clinical trial, which used only a 6-week IMST intervention (Craighead et al., 2021b). BP continued to decline throughout the intervention period, supporting the possibility that a longer treatment duration would result in further reductions in BP. Overall, our new trial will allow us to examine the magnitude of improvement in cardiovascular function following a longer treatment duration and to assess the safety, tolerability, and adherence profiles of high-resistance IMST over an extended period.

As all previous studies on high-resistance IMST have used a low-resistance sham control group, our new clinical trial will, for the first time, compare IMST to guideline-based aerobic exercise training, thus allowing us to directly assess the relative effectiveness of IMST compared with “standard of care” aerobic exercise. The innovative study design will enable direct comparisons of the safety, tolerability, adherence, efficacy and underlying molecular mechanisms between high-resistance IMST and aerobic exercise.

Finally, this current trial features additional outcome measures not assessed in any previous trial on high-resistance IMST. These outcomes include *in vivo* assessment of reactive oxygen species-associated suppression of vascular endothelial function, biopsied endothelial cell protein markers of oxidative stress, vascular smooth muscle sensitivity to NO, and *ex vivo* endothelial cell production of NO and ROS following administration of serum or other experimental manipulations to provide insight into mechanisms of action. In addition, along with casual SBP, the persistent effects of IMST and aerobic exercise on 24-h SBP and vascular endothelial function following the cessation of the respective interventions will be measured. Overall, there are a significant number of novel and innovative aspects of this trial that will provide important new information regarding high-resistance IMST as a potential healthy lifestyle intervention.

## Conclusion

This clinical trial is an essential next step from our small pilot study for the translation of the cardiovascular benefits of high-resistance IMST towards implementation in clinical practice and public health. This trial also addresses an important research gap in the understudied area of women’s cardiovascular health by investigating an evidence-based lifestyle intervention for preserving endothelial function with aging in estrogen-deficient postmenopausal women. If our hypotheses and expected results are confirmed, it will provide a platform for investigating the potential of IMST to improve cardiovascular health in other groups of women at increased risk for CVD, such as those with endometriosis or a history of gestational hypertension. Finally, our expected results on a low-barrier intervention could be translated to urban and rural populations subject to health disparities who lack access to fitness centers and built environments conducive to conventional aerobic exercise training or the financial means to purchase expensive home exercise equipment.
